# Transcriptomic research in atherosclerosis: Unravelling plaque phenotype and overcoming methodological challenges

**DOI:** 10.1016/j.jmccpl.2023.100048

**Published:** 2023-09-12

**Authors:** Miron Sopić, Kanita Karaduzovic-Hadziabdic, Dimitris Kardassis, Lars Maegdefessel, Fabio Martelli, Ari Meerson, Jelena Munjas, Loredan S. Niculescu, Monika Stoll, Paolo Magni, Yvan Devaux, CardioRNA COST Action CA17129 and AtheroNET COST Action CA21153

**Affiliations:** a Department of Medical Biochemistry, Faculty of Pharmacy, University of Belgrade, Luxembourg Institute of Health, L-1445 Strassen, Luxembourg; b Cardiovascular Research Unit, Department of Population Health, Luxembourg Institute of Health, L-1445 Strassen, Luxembourg; c Faculty of Engineering and Natural Science, Computer Science, International University of Sarajevo, Bosnia and Herzegovina; d Laboratory of Biochemistry, University of Crete Medical School and Gene Regulation and Epigenetics Group, Institute of Molecular Biology and Biotechnology, Foundation for Research and Technology of Hellas, Heraklion 71003, Greece; e Institute of Molecular Vascular Medicine, Klinikum rechts der Isar, Technical University Munich, Germany; f German Center for Cardiovascular Research (DZHK), partner site Munich Heart Alliance, Department of Medicine, Karolinska Institute, Stockholm, Sweden; g Molecular Cardiology Laboratory, IRCCS-Policlinico San Donato, via Morandi 30, 20097, San Donato Milanese, Milan, Italy; h Molecular Biology of Chronic Diseases Laboratory, Genomic Center, Galilee Research Institute (MIGAL), Kiryat Shmona, Israel; i Faculty of Sciences, Tel Hai Academic College, Israel; j Department of Medical Biochemistry, Faculty of Pharmacy, University of Belgrade, Serbia; k Lipidomics Department, Institute of Cellular Biology and Pathology “Nicolae Simionescu” of the Romanian Academy, 8, B.P. Hasdeu Street, Bucharest 050568, Romania; l University of Münster, Institute of Hunan Genetics, Division of Genetic Epidemiology, Münster, Germany; m Maastricht University, Dept. of Biochemistry, Genetic Epidemiology and Statistical Genetics, Maastricht, NL; n Department of Pharmacological and Biomolecular Sciences “Rodolfo Paoletti”, Università degli Studi di Milano, via Balzaretti 9, 20133, Milan, Italy; o IRCCS MultiMedica, via Milanese 300, 20099 Sesto S. Giovanni, Milan, Italy

**Keywords:** Atherosclerotic plaque, Transcriptomics, Data integration, machine learning

## Abstract

Atherosclerotic disease is a major cause of acute cardiovascular events. A deeper understanding of its underlying mechanisms will allow advancing personalized and patient-centered healthcare. Transcriptomic research has proven to be a powerful tool for unravelling the complex molecular pathways that drive atherosclerosis. However, low reproducibility of research findings and lack of standardization of procedures pose significant challenges in this field. In this review, we discuss how transcriptomic research can help in understanding the different phenotypes of the atherosclerotic plaque that contribute to the development and progression of atherosclerosis. We highlight the methodological challenges that need to be addressed to improve research outputs, and emphasize the importance of research protocols harmonization. We also discuss recent advances in transcriptomic research, including bulk or single-cell sequencing, and their added value in plaque phenotyping. Finally, we explore how integrated multiomics data and machine learning improve understanding of atherosclerosis and provide directions for future research.

## Highlights

• A deep characterization of the molecular mechanisms responsible for atherosclerotic plaque development is needed.
• RNAs and particularly non-coding RNAs regulate gene expression and play a major role in atherosclerotic plaque development.
• Methodological challenges in transcriptomic studies hinder the translation of basic research findings to clinics.
• Tips and tricks are provided to improve translational capacity of basic transcriptomic research for patient’s benefit.

## Graphical abstract

**Figure:**
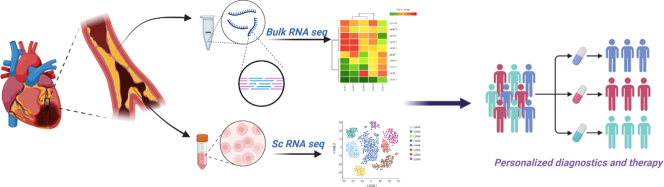

## 1 Introduction

According to 2022 Heart Disease & Stroke Statistical Update Fact Sheet Global Burden of Disease, approximately 19.1 million deaths were caused by cardiovascular disease (CVD) worldwide [1]. Major part of all CVDs is ischemic heart disease (IHD) that currently affects 244.1 million people globally, with mortality rates 112.37 per 100,000 [1]. Hence, despite significant improvements of healthcare procedures and patient outcomes, CVDs still remain a major public health issue with a high socio-economic burden. Ischemic heart disease is characterized by the narrowing or blockage of major coronary blood vessels, which is primarily caused by atherosclerotic plaques. Most acute coronary events are triggered by plaque rupture (majority of all ACS, 73 %) or plaque erosion, followed by coronary thrombosis and obstruction of the coronary blood vessels [2]. Overall, plaque composition, plaque stability, and its interaction with the vascular microenvironment are the main factors that determine the development of acute coronary events. The heterogeneity of pathophysiological mechanisms of plaque destabilization is reflected in the complexity of clinical presentations of ACS [3]. However, the traditional diagnostic classification of ACS does not reflect this disparity. Despite recent efforts, there are still no reliable biomarkers reflecting the underlying mechanism that yields acute ischemia [3]. There is a gap between plaque complexity and our ability to recognize different plaque phenotypes in the clinical setting. Thus, new tools are needed to improve our understanding of plaque pathophysiology, which can be translated from the bench to the bedside and help prevent poor outcomes in ACS.

An important level of complexity in cellular signalling is related to dynamic changes in gene expression. Mounting evidence suggests that these dynamic changes in the transcriptome accompany pathological processes in many organs. In the past decade, technological advancements and the use of next-generation sequencing enabled comprehensive transcriptome analysis, deepening our understanding of RNA complexity. Special attention has been drawn to non-coding RNAs (ncRNAs), the significance of which is illustrated by the fact that only ∼2–3 % of human transcriptome consists of protein-coding RNAs or messenger RNAs (mRNAs), and over 90 % of ncRNAs [4]. NcRNAs contribute to eukaryotic complexity by regulating gene expression. NcRNAs such as microRNAs (miRNAs), long non-coding RNAs (lncRNAs) and circular RNAs (circRNAs) are involved in the progression of atherosclerosis by affecting vascular endothelium, inflammation and cholesterol metabolism. Detailed description of these regulatory mechanisms is available [[5], [6], [7]]. Considering the cell-specific effects that ncRNAs can exert on cellular functions and pathophysiological signalling pathways related to atherosclerosis, it is likely that these molecules represent targets for future therapies or biomarkers for personalized healthcare and precise diagnosis of atherosclerotic CVD. However, multiple methodological challenges hinder the translation of findings from basic transcriptomics research to clinical application. In this review, we underline these challenges and propose tips and tricks for the design of future studies with the ultimate goal to improve translational research and patient's outcomes.

## 2 Transcriptomic research in atherosclerosis

### 2.1 Human studies

Advanced atherosclerosis samples are typically obtained during surgeries like carotid endarterectomies (CEA) or from autopsies. While carotid and leg artery samples are common, coronary artery tissues, rarely sampled during procedures, provide insights into plaque development and myocardial infarction.

Various approaches were pioneered to identify drivers of plaque progression and instability, as well as lesions' ability to cause clinical symptoms like myocardial infarction, stroke, or ischemia to the periphery (most commonly the lower extremities). Several groups have chosen to assess symptoms of patients as their main criteria [8,9]. Importantly, the AtheroExpress consortium collected follow-up data from patients that underwent CEA to identify predictive markers of future cardiovascular events in individuals with atherosclerotic disease [10,11]. For this type of analysis, symptomatic patients are compared to asymptomatic patients. This comparison has proven quite powerful when identifying plaques from patients with a more aggressive atherosclerotic phenotype. These findings could often be linked to mechanisms involved in immune cell infiltration, vessel wall inflammation [12,13], as well as arterial remodelling which involves SMC (de)differentiation [14].

Apart from studying atherosclerosis from a clinical perspective (comparing symptomatic to asymptomatic patients), differential gene expression analysis was performed based on changes in the plaque phenotype. Thus, stable plaques (thicker than 200 μm) were compared with unstable and/or ruptured plaques (thinner than 200 μm) [15,16]. The biggest advantage of this type of comparison is the assessment of plaque phenotype in addition to evaluating patients' symptoms. However, it is challenging to characterize the plaque phenotype in addition to harvesting material of sufficiently high quality and quantity to extract RNA for transcriptomics analysis which are sensitive to RNA degradation (e.g., RNA sequencing, PCR).

In addition to analysing whole plaque tissue specimens, researchers have performed micro-dissection laser capture to determine the profile of distinct plaque areas, such as the necrotic core or the fibrous cap [17,18]. These methods enable one to determine specific RNA profiles of areas directly relevant to plaque stability, such as the fibrous cap which serves as the last line of defence by stabilizing the lesion.

One issue affecting all studies involving human patient material is the lack of un-diseased, non-atherosclerotic vascular control tissue. Specimens from the internal mammary artery (IMA), which is commonly used for coronary arterial bypass graft (CABG) surgery; or iliac arteries from patients undergoing open surgical repair for an abdominal aortic aneurysm may be useful in that respect [19,20]. One issue with all these types of arterial tissues is that their composition (muscularity), the number of elastic layers, and exposure to alternating flow and shear stress patterns differs from coronary of carotid arteries, which are usually analysed in human atherosclerosis. Their embryonic origins also differ, reflected in differences in structural or mural cells like endothelial or smooth muscle cells, as well as adventitial fibroblasts [21].

Vulnerable plaque is a term that refers to unstable plaques that are susceptible to the physical disruption that leads to thrombosis, the formation of blood clots and heart attack or stroke. However, the process of plaque formation and rupture is complex and multifactorial, and no single plaque phenotype is consistently associated with an increased risk of rupture. Insights from transcriptomic studies facilitated the discernment of subtle variations between plaques. These studies unveil new determinants of plaque destabilization with potential clinical utility.

To establish connections between global transcriptomic profiles and clinical features that stratify stroke risk, Matic et al., used transcriptomic analysis to explore correlations between carotid atherosclerosis phenotypes and gene expression patterns [9]. The study identified subtle gene expression distinctions between symptomatic and asymptomatic plaque patients, underscoring the role of gene coordination in managing these disparities. Notably, more pronounced gene expression variations were associated with the time elapsed between symptoms and surgery, along with statin therapy. Statin-treated patients exhibited pathways linked to stable plaques, such as angiogenesis suppression and matrix metalloproteinase inhibition. Interestingly, statins also promoted calcification and inhibited tissue resorption. Additionally, plaque biology changed after rupture, involving swift tissue repair followed by prolonged immune responses. This suggests that plaque composition is influenced not only by atherosclerosis progression but also by the locations of blood vessels. These findings shed light on the complex interplay of factors shaping plaque stability and vulnerability. Furthermore, it seems that plaque composition is not solely dependent on the progression of atherosclerosis, but also on locations of blood vessels. Using transcriptomics, Steenamn et al. found a major difference between femoral and carotid atherosclerotic plaques, with femoral plaques enriched in genes involved in osteoblast differentiation and bone morphogenesis [22]. Carotid plaques were more enriched in immune response and lipid metabolism genes, suggesting that arterial heterogeneity may determine susceptibility to fibrosis and calcification. The carotid arteries are more prone to microcalcifications, while osteoid metaplasia is more common in the femoral arteries. Interestingly, the authors showed that traditional cardiovascular risk factors are not associated with the type of calcification, but with the risk of progression of atherosclerosis in peripheral arteries.

The hyperplasia and migration of smooth muscle cells (SMCs) from the media to the intima of the arterial wall inevitably leads to destabilization of the plaque. Notably, recent advancements have significantly deepened our understanding of this process. Rykaczewska et al. applied innovative integration of plaque evaluation with ultrasound and transcriptomics and unveiled a direct link between carotid plaque echogenicity and distinct molecular signatures involving calcification, iron homeostasis, cell survival, and SMC transdifferentiation [23]. In particular, their study uncovered the previously unknown significance of BCL2-associated transcription factor 1 (BCLAF1) that emerged as a critical regulator repressed in echolucent, lipid-rich carotid plaques that are typically associated with a vulnerable patient phenotype and heightened cardiovascular risk. Functionally, BCLAF1 is vital for SMC survival and lipid-induced transdifferentiation into macrophage-like cells within the plaque, regulating genes including Krüppel-like factor 4 (KLF4), BCL2, cluster of differentiation (CD36), and cluster of differentiation (CD68). Additionally, transcriptomic analysis of SMCs from carotid plaques revealed more senescent phenotype in SMCs from symptomatic plaques and an osteogenic phenotype in SMCs from asymptomatic plaques, while TGF-β signalling was identified as the mechanism defining SMC phenotype, suggesting a role in plaque destabilization [24]. Alsaigh et al. compared transcriptome profiles of calcified atherosclerotic plaques in the core and proximal sections using single-cell RNA sequencing (scRNA-seq), showing that SMCs and endothelial cells are involved in calcification and extracellular matrix remodelling [25]. Depuydt et al. observed that My.2 macrophages express SMC actin, suggesting derivation from SMCs [26]. ScRNA-seq and sex-specific gene regulatory networks also suggest differences in SMC biology which play a role in sex-specific pathophysiology of atherosclerosis [27].

Recent studies using single-cell transcriptomics gave deeper insights into plaques heterogenic content of different immune cells, endothelial cells, smooth muscle cells etc. [[28], [29], [30]], and even show proof of endothelial to mesenchymal transition and the gene expression profile of these trajectories in human atherosclerotic plaques [30]. ScRNA-seq of human atherosclerotic lesions revealed T cells and macrophages as the most abundant populations, with disrupted plaques in symptomatic patients enriched in effector-memory CD4+ T cells and distinctive macrophage phenotypes [31]. The authors found increased expression of PD-1, a marker of T cell exhaustion, in T cells present in atherosclerotic plaques of patients with recent acute events. Inhibiting PD-1 may activate exhausted T cells and worsen atherosclerosis, potentially causing unpredictable adverse effects in cancer patients with underlying CVD [32]. Depuydt et al., used scRNA-seq and scATAC-seq on carotid plaques and identified 14 cell clusters, including 11 immune and 3 non-immune clusters [26]. The authors integrated GWAS and scRNA-seq data to identify specific cell populations involved in CVD susceptibility loci, suggesting that this approach may be useful for pinpointing drug targets for personalized intervention. Moreover, scRNA-seq technology has illuminated novel macrophage phenotypes within atherosclerotic plaques. For instance, CD136^+^ macrophages clustered around intraplaque microvessels, displaying elevated hypoxia-inducible factor 1-alpha (HIF1α) and vascular endothelial growth factor A (VEGF-A) expression associated with plaque progression and angiogenesis [33]. These macrophages exacerbated plaque inflammation by increasing vascular cell adhesion molecule (VCAM) expression in intraplaque endothelial cells. Another recent addition to the macrophage repertoire, as elucidated by Karamanavi et al., is the FES^+^ (FES proto-oncogene) macrophage phenotype, with potential atheroprotective properties [34].

By combining scRNA-seq of atherosclerotic tissue with sex-specific gene regulatory networks, Hartman et al. revealed that women had higher gene activity associated with mesenchymal and endothelial cells, while men had higher activity associated with the immune system [27]. Jin et al. showed that sex-specific genes correlate with both biological sex and plaque phenotype, notably involving fibrosis and inflammation pathways rather than sex-hormone responses [35]. Male- and female-specific key drivers were significantly associated with unstable-specific genes, suggesting a connection to biological sex, potentially due to shared common genes. Functional pathway analysis revealed JAK-STAT predominance in unstable plaques and profibrotic EGFR and TGFβ pathways in stable plaques. Additionally, distinct patterns emerged in inflammation and profibrotic pathways within the intersects of unstable-male and stable-female genes. Unexpectedly, androgen and estrogen pathways showed opposing associations with these intersects. Genes driven solely by biological sex exhibited enrichment in catecholamine secretion and muscle cell contraction terms, revealing sex-dependent processes and underlining the importance of sex differences in atherosclerosis mechanisms.

Although scRNA-seq gives deep insights into cellular composition of atherosclerotic plaques, it is limited in its inability to provide information about the spatial location of cells within a plaque. In the study by Sun et al. gene expression signatures in distinct regions of human carotid plaques were analysed using various techniques including histology, scanning electron microscopy, RNA sequencing, and spatial transcriptomics [36]. By focusing on longitudinal blood flow direction (proximal, most stenotic, and distal regions), the researchers revealed that plaque ruptures rarely emerge distally to the most stenotic region, uncovering a unique gene expression pattern associated with atherosclerosis-related diseases. This spatial transcriptomics approach not only broadened the understanding of molecular mechanisms underlying plaque rupture but also allowed the localization of specific gene expressions within plaque tissue sections, such as MMP9, immunoglobulin kappa constant, and phospholamban. This technique revealed that MMP9 was prominently expressed in unstable shoulders, a common rupture site, and showed co-localization with integrin alpha X (ITGAX), a marker for proinflammatory macrophages, contributing to the formation of rupture-prone plaques.

Transcriptomic signatures of atherosclerotic plaques can also provide valuable clinical information. Mokry et al. performed a transcriptomic analysis of 654 advanced human carotid plaques and were able to distinguish five dominant types of plaque [37]. Plaques with the most severe clinical symptoms expressed a number of genes related to inflammation, neutrophil degranulation, matrix changes and metabolism. The results were validated in 162 samples of coronary artery plaques confirming that the fibro-inflammatory type of plaque was strongly associated with coronary ischaemic events. Transcriptomics can also complement imaging techniques to provide a holistic view of patient conditions. Integrating molecular insights with carotid computed tomography angiography (CTA) enables linking imaging biomarkers to underlying pathophysiological processes [38]. Advancing this approach, recent investigations harnessed computer-based CTA image analysis to define plaque morphology, forging correlations between visual characteristics, symptomatology, and molecular profiles from corresponding carotid endarterectomy (CEA) samples [38]. This approach pioneered a profound connection between carotid plaque morphology and biological traits, shedding light on the intricate interplay between appearances and underlying mechanisms. Furthermore, the emergence of “virtual transcriptomics” as a concept that bridges gene expression insights with non-invasive imaging data augments the potential for enhanced clinical applications, promising a more comprehensive understanding of disease processes and patient-specific needs. This concept was presented by Buckler et al. that employed machine intelligence to integrate CTA images and transcriptomics from carotid endarterectomies of 40 patients with carotid stenosis [39]. Their innovative approach involved novel software to analyse CTA images for precise plaque morphology characterization and extraction of plaque transcriptomes from microarrays. Mathematical modelling predicted gene expression patterns, identifying 414 coding and noncoding RNAs linked to plaque morphology. The models' predictive accuracy was validated using CTA images from separate patients and corresponding transcriptomes. Thus, transcriptomic characterization of plaques can provide valuable data that can be used for better stratification of patients, improved diagnosis and tailored treatments.

### 2.2 Animal studies

Despite significant differences related to their plasma lipid and lipoprotein profiles and their vascular anatomy relative to humans, small rodents have been used extensively in atherosclerosis studies for various reasons: a) short life cycle, b) large numbers of progeny produced many times a year, c) easy in experimentation, d) low cost, e) easy genetic manipulation, f) plethora of strains with different genetic backgrounds and predisposition to various diseases. Mice are resistant to development of advanced atherosclerotic plaques even after feeding with Western diets, mainly due to absence of atherogenic lipoproteins such as low-density lipoproteins (LDL) in their plasma [40]. Contrary to humans, the main circulating lipoprotein in the mouse plasma is high density lipoproteins (HDL) due to the lack of expression of the gene encoding the plasma lipid transfer protein Cholesteryl Ester Transfer Protein (CETP) which transfers cholesteryl esters from HDL to very low density lipoproteins (VLDL)/LDL in exchange for triglycerides [41]. The increase of HDL in the plasma confers atheroprotections due to its many anti-atherogenic functions including reverse cholesterol transport, anti-inflammatory, anti-oxidant, anti-thrombotic and many others [42].

Two mouse models that have been used extensively for atherosclerosis translational research are mice lacking the LDL receptor (LDLR^−/−^ mice) and mice lacking apolipoprotein E (apoΕ^−/−^ mice). In LDLR^−/−^ mice (mimicking familial hypercholesterolemia in humans), mild atherosclerotic lesions develop on a chow diet but strong acceleration in plaque development is observed on a western type diet [43]. The limitations of this model is that plaques develop primarily in the aorta (not in coronary arteries as in humans) and that plaques do not rupture and lead to thrombosis. ApoΕ−/− mice do not express apolipoprotein E (apoE), a 34 kDa protein that is produced by many tissues including the liver, by macrophages and the brain and associates with lipoprotein particles such as VLDL, chylomicrons and HDL facilitating their clearance via the LDL receptor [43]. Although this model has been widely used for understanding the pathophysiology of atherosclerosis, it has several limitations compared to humans with the most notable being the absence of thrombotic occlusion in the coronary arteries causing MI. This could be due to anatomical differences such as smaller vessels and lower surface tension compared to the humans [44]. Plaque rupture rarely occurs in this model and only in the brachiocephalic arteries and the aorta. Mice models with plaques that are more close to human atherosclerotic lesions can be generated with genetic perturbations including a mutant form of fibrillin 1 (Fbn1 ^C1039G+/−^) [45] or expressing a combination of the human apoE*3 Leiden mutant (apoE*3-Leiden) and CETP genes [46]. An alternative, less complicated and easier to generate, mouse model of atherosclerosis that has been developed recently is the administration of adeno-associated viruses (AAVs) expressing human Proprotein convertase subtilisin/kexin type 9 (PCSK9) [47]. PCSK9 is a serine protease that is secreted by the liver and other tissues and binds strongly to the LDL receptor in the endosomes preventing its recycling and reducing its life cycle [48]. Overexpression of PCSK9 via AAVs in combination with the administration of a high fat diet and partial carotid ligation caused significant reduction of the LDLR on the plasma membrane in the liver and accumulation of LDL in plasms, similarly to the LDLR −/− mice [49]. A serious drawback of the mouse models described above is the absence of atherosclerosis in the coronary arteries, which is a characteristic clinical feature in humans. To overcome this problem and achieve coronary atherosclerotic plaque development, investigators generated mice with double deficiency in both the apoE and the LDLR genes (LDLR^−/−^/apoE^−/−^ mice). These mice developed atherosclerosis in the aorta and coronary arteries after feeding with a fat diet. They also suffered from MI which is a rare clinical characteristic in mouse models of atherosclerosis [50]. Coronary atherosclerosis was also achieved in a series of mouse models that combined deficiency of either the LDLR or the apoE genes with the gene encoding the HDL receptor SR-BI (Scavenger receptor Class B Type 1) [51,52], or genes that interact with or are activated by SR-BI in endothelial cells such as the endothelial nitric oxide synthase (eNOS) [53] and the PDZ containing 1 (PDZK1) [54].

Yang et al. [55] studied the atherosclerotic plaque transcriptome in wild-type mice fed a chow diet and in apoE−/− mice fed with a Western type diet for 20 weeks. They identified 96 differentially expressed genes (DEGs) in advanced atherosclerotic plaque compared with early plaque and 838 DEGs in ruptured atherosclerotic plaque compared with stable plaque. Using bulk and scRNA-seq, Kim et al. [56] showed that non-foamy rather than foamy macrophages have pro-inflammatory roles in apoE^−/−^ and LDLR^−/−^ mice. Using single cell transcriptomics, Winkels et al. characterized leukocytes from chow diet- and Western diet-fed Apoe−/− and Ldlr−/− mice aortas in order to generate an immune cell atlas of atherosclerotic plaques [57]. They detected 11 principal leukocyte clusters with distinct phenotypic and spatial characteristics. Gene set enrichment analysis showed that lipid metabolism, proliferation, and cytokine secretion were confined to specific clusters. Phenotypic switching is a pathological process in which smooth muscle cells (SMCs) dedifferentiate, migrate, and transdifferentiate into other cell types and contribute to atherosclerosis. This mechanism was studied at the single cell level by Pan et al. by combining SMC fate mapping and scRNA-seq of mouse atherosclerotic plaques [58]. The authors showed that SMCs transitioned to an intermediate cell state during atherosclerosis termed “SEM” (stem cell, endothelial cell, monocyte)] in which they were multipotent and could differentiate into macrophage-like and fibrochondrocyte-like cells. The method of laser capture microdissection (LCM) allows the isolation of individual cell populations from tissue sections with the use of lasers and combined with RNA amplification (due to small RNA concentration in the captured samples) and microarray techniques has been applied to measurement of gene expression in atherosclerotic lesions in mice [59,60].

Rats are more convenient models for atherosclerosis research due to their larger size compared to mice, but their major drawback is complexity of genetic manipulation. Recently, using the zinc finger technology, LDLR−/− rats have been generated that develop extended atherosclerosis only after the administration of Paigen diet [61] for a long period (50 weeks) [62]. The same technology was used to generate apoE −/− rats which develop extended atherosclerosis with the administration of a Paigen diet for 64 weeks [63].

Rabbits develop atherosclerosis faster than other experimental animals, mostly due to the absence of hepatic apolipoprotein B expression [64]. Watanabe-heritable hyperlipidemic (WHHL) rabbit develop aortic and coronary atherosclerosis. The aortic lesions are characterized by ample intimal thickening, foam cells and a fibrotic component, a histologic appearance similar to human lesions ([65,66]. Transcriptomic analyses on rabbit atherosclerotic lesions identified network of several lncRNAs and miRNAs that could be used as prospective biomarkers of atherosclerosis progression [67].

The Golden Syrian hamster presents many similarities with the human lipid metabolism. At more advanced stages of atherosclerosis, the plaques in hamsters are characterized by accumulation of extracellular unesterified cholesterol, calcium deposition and necrosis. Similar to humans, male hamsters are being more susceptible to an atherogenic diet than females. Recently, Barbalata et al. performed a microarray miRNA profiling in tissues of hyperlipidemichamsters. They reported a dysregulation of miRNAs expression with a discrete distribution in the heart-liver axis [68].

**Fig. 1:**
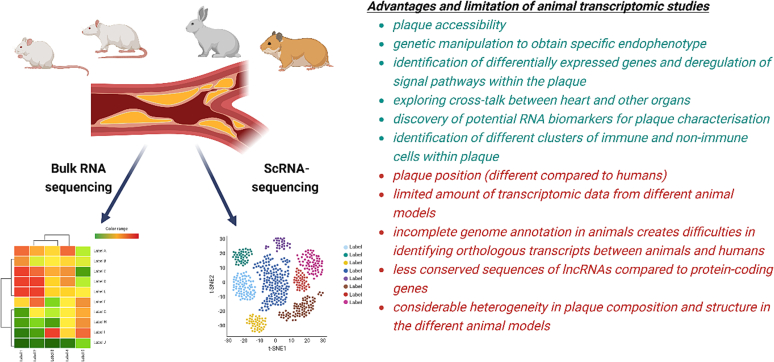
The translational potential of animal transcriptomics studies (advantages are presented in green and limitations in red) (Created with BioRender). (For interpretation of the references to colour in this figure legend, the reader is referred to the web version of this article.)

There are several limitations that limit the translatability of mouse model transcriptomics in atherosclerotic coronary heart disease. These limitations include: a) incomplete genome annotation in mice [69,70]; b) less conserved sequences of lncRNAs compared to protein-coding genes [71]; c) lncRNAs are usually expressed at lower level than protein-coding genes and often in a very tissue-specific manner [71,72]; d) there is considerable heterogeneity in plaque composition and structure in the different mouse models and in very few models rupture-prone plaques can be found, in contrast to human ASCAD patients, where plaque rupture is common. The main challenges in the translational potential of animal transcriptomics studies are summarized at Fig. 1.

## 3 Methodological challenge in transcriptomic research

### 3.1 Bulk RNA sequencing

**Fig. 2:**
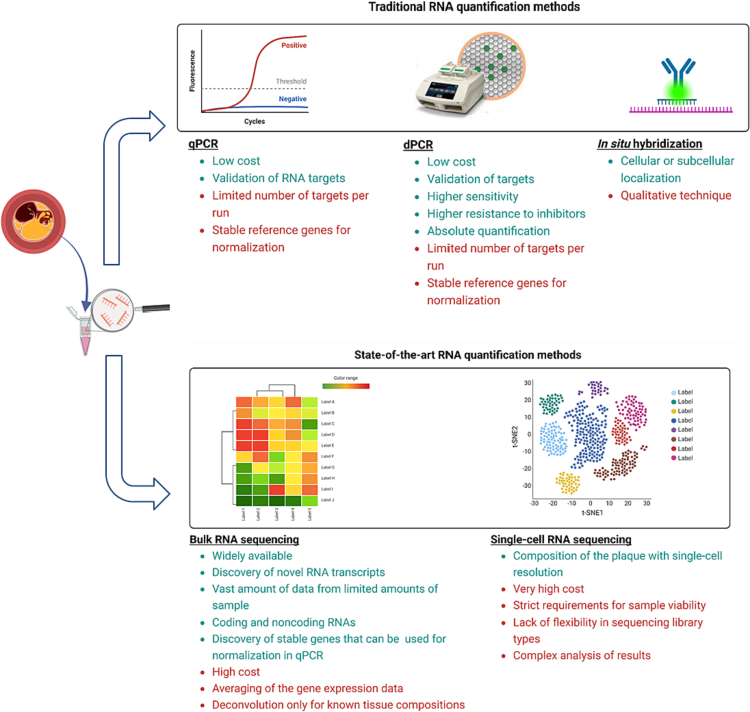
Advantages (green) and limitations (red) of traditional and novel RNA quantification methods. (Created with BioRender). (For interpretation of the references to colour in this figure legend, the reader is referred to the web version of this article.)

RNA extraction from atherosclerotic plaques can be challenging due to their highly necrotic and acellular nature, as well as increased levels of reactive oxygen species (ROS) caused by inflammation and intra-plaque haemorrhage [73]. Classical phenol-based methods or silica column isolation kits can be used for RNA purification, but contaminating agents such as heparin must be considered [74]. RNA quantification and quality checks performed using spectrophotometry, electrophoresis, and RNA integrity number (RIN) values are essential steps prior to downstream analysis methods [75]. More basic techniques such as RT-qPCR, digital PCR and in situ hybridisation techniques are still being extensively used for RNA quantification. Major limitations and advances of these techniques are presented on the Fig. 2. However, in this review we will keep focus on more advanced and comprehensive methods.

In the past decade, bulk RNA sequencing has become the standard tool for identifying and quantifying changes in transcript levels in a variety of biological contexts. It has, for the first time, enabled a truly hypothesis-free analysis of a RNA sample (prior screens usually relied on microarrays, where discovery of biological effects was limited to the existing probe set). By now, a variety of specific protocols has been developed in order to target particular classes of RNAs with Illumina-type libraries. Before sequencing, proper library preparation is crucial, including target enrichment. To avoid overrepresentation of abundant ribosomal RNAs (rRNAs) and reduce sequencing depth, rRNA transcripts should be removed [76]. Alternatively, poly(A) enrichment can be used for sequencing protein-coding RNAs and some poly(A)-tailed lncRNAs [77]. However, rRNA-depletion is better suited for ncRNA research as it allows quantification of ncRNAs without a poly(A) tail, including pre-mRNA and circular ncRNAs [77]. Recent advances in protocols that enable simultaneous RNA sequencing of the small and long transcripts from the same total RNA extract enable a more comprehensive analysis of transcriptome [78]. In the context of atherosclerotic plaque research, one of the limiting factors is related to the availability of the proper samples that could be used for RNA sequencing. Indeed, human plaques are very hard to obtain, especially those from coronary arteries. The final result depends on the origin of the plaque and the amount of surrounding tissues sampled through the procedure. On the other hand, the sensitivity of RNA sequencing enables the generation of comprehensive data from a small and limited amount of sample. In addition, RNA sequencing can be used for the discovery of stably expressed genes to use as housekeeping genes [79]. Housekeeping genes can also be identified using Genorm or Normfinder [79], and the most stable gene(s) can be utilized for the normalization of qPCR data. In addition to sequencing by synthesis methods, Oxford Nanopore Technologies developed method for direct RNA sequencing. This technology enable very long reads (tens and even hundreds of kilobases in length, albeit with overall higher error rates than short reads) and detection of RNA modification.

Bulk RNA sequencing can overlook cellular heterogeneity and interactions in complex systems such as atherosclerotic plaques, resulting in averaged gene expression data that fails to provide a complete picture of tissue transcriptome complexity. Deconvolution methods can help dissect the cellular components found in a bulk transcriptome, but currently require prior knowledge of tissue composition [80]. Single cell RNA sequencing (scRNA-seq) can identify gene signatures necessary for accurate deconvolution, allowing previously generated bulk RNA sequencing data to be deconvoluted. Efforts are underway to develop deconvolution methods that do not require prior tissue composition knowledge [28,81].

### 3.2 ScRNA-seq

Gene expression variation among cells in a bulk sample masks essential changes, necessitating the assessment of individual cell transcriptomes. Microfluidic devices compartmentalize single cells prior to RNA-seq library preparation, with conventional RNA-seq methods. scRNA-seq systems have existed for about 5 years, enabling new studies and data availability from different biological contexts. Recently, a method was developed for sequencing small amounts of RNA from live cells without lysing them, enabling longitudinal single-cell transcriptomics [82]. The main challenges in the wider use of scRNA-seq technologies include:

#### 3.2.1 Costs

scRNA-seq cost is a major barrier to adoption by healthcare providers, as it is an order of magnitude more expensive than bulk RNAseq. However, new microfluidic devices with more reagent flexibility and multi-omic data collection are being developed, and as they become more widely adopted, costs are expected to decrease [83,84].

#### 3.2.2 Cell viability

10× Chromium protocols previously required high cell viability and limited sample types for scRNA-seq. New developments include the 10× Visium CytAssist, enabling scRNA-seq of FFPE tissue samples and designed for spatial transcriptomics.

#### 3.2.3 Flexibility

Proprietary commercial kits for scRNA-seq focus on capturing polyA+ mRNA, excluding small RNAs, tRNAs, and lncRNAs. Open protocols and new companies entering the field may resolve this issue. Long-read single-cell sequencing is also possible with 10× cell compartmentalization and Oxford Nanopore sequencing [85].

#### 3.2.4 Complexity of data

scRNA-seq data analysis is complex due to the presence of heterogenous subsets of transcripts representing single cells in a multi-dimensional space of expression data, batch effects, and the need for identifying specific cell clusters. However, new software tools are addressing these challenges, which will likely consolidate and become more user-friendly in the future.

## 4 Deep plaque phenotyping through integration of transcriptomics with other omics approaches (multiomics approach)

In the past several years the technologies for different omics analysis have become more available, which led to combined omics (genomics, *epi*genetic, (epi)transcriptomic, proteomic, metabolomic) analysis in the same set of samples. By using this so-called multiomics approach and integrating all the data, it is now possible to put transcriptomic data into wider biological context and discover additional levels of complexity. In the study by Matic et al., this integrative approach was developed to identify biomarkers for carotid atherosclerosis by analysing transcriptomic and proteomic profiles of plaques and plasma from patients with carotid stenosis undergoing surgery [86]. Biliverdin reductase B (BLVRB) was found to be enriched in both plaques and plasma of patients with carotid atherosclerosis, particularly associated with intraplaque haemorrhage (IPH). This discovery introduces BLVRB as a potential biomarker for identifying end-stage vulnerable plaques and individuals at higher risk of stroke. Building upon this approach, Jin et al., performed an integrative analysis to construct a predictive model distinguishing low- and high-risk carotid artery lesions by utilizing transcriptomic, proteomic, and peptidomic profiles from human carotid artery plaques [87]. The study demonstrated the effectiveness of this model across independent plaques and highlighted the role of serum response factor-driven regulatory gene/protein network in peri-rupture remodelling, particularly associated with intimal processes related to intraplaque haemorrhage.

**Fig. 3:**
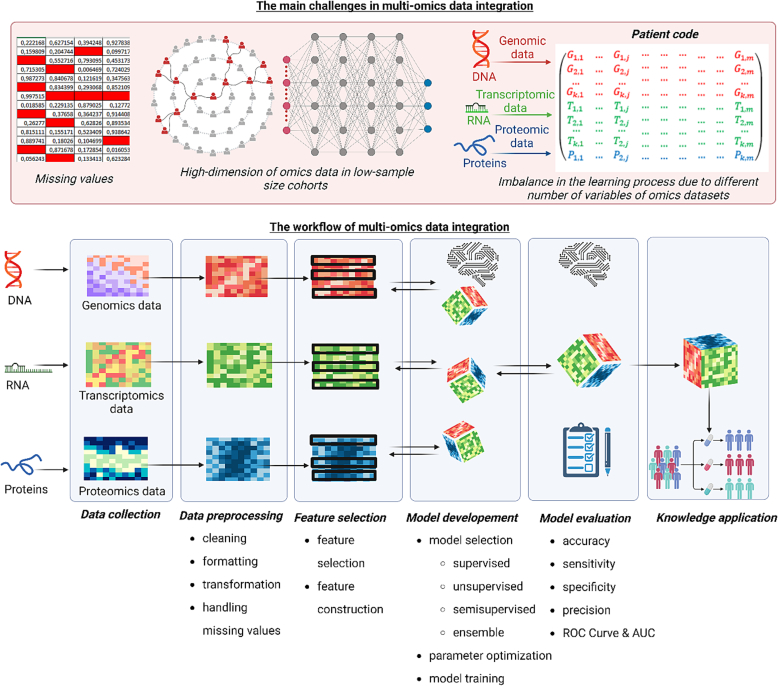
Using machine learning for integration of transcriptomic with other omics data. (Created with BioRender).

In order to extract relevant information from multiomics data, advanced computational techniques such as machine learning have become an important tool in biomedical data analysis. Machine learning is a subfield of artificial intelligence with the main aim to iteratively learn from data in order to identify complex patterns and insights without explicitly being programmed to do so. The machine learning pipeline starts with the collection of data from various sources (e.g. multiomics data, clinical data). Afterwards, data preprocessing is performed which includes data cleaning, data integration, formatting, transformation, etc. This is followed by the feature selection process. Dataset is then split into distinct training and test datasets. Training dataset is used for model development which includes selecting and building the best machine learning model. Afterwards, performance of the model is evaluated using the test dataset, and if necessary, model performance is improved. Finally, the acquired knowledge is ready to be applied to the problem at hand (Fig. 3).

Even though machine learning has been applied to analyse a variety of biomedical data, it has not yet been widely used to analyse transcriptomics data. However, its use is constantly expanding, largely because of the large amount of availability of transcriptomic data and the ability of machine learning algorithms to uncover intricate relationships within the data by complementing conventional statistical approaches of transcriptomic data analysis. So far, machine learning has been used for the analysis of transcriptomic data including in Parkinson's diseases [88] and colorectal cancer [89]. In the context of CVD, machine learning enabled better classification and prediction of atherosclerotic disease based on the large number of clinical data and blood-based biomarkers [90,91].

More so than its usage in transcriptomic research, application of machine learning to multiomics data is still in its infancy. As outlined in [92], one of the main reasons for this are the challenges of integrating multiomics datasets such as the occurrence of missing values and class imbalance, which are common problems to many machine learning datasets. Others are specific to biological data such as to the noisiness and complexity of omics datasets, and high-dimension, low-sample size problem, a problem that often leads to building a machine learning model that does not generalize well on the new unseen data. Omics data may also vary in data dimensionality or data types such as numerical, categorical, continuous, discrete, etc., which must be properly managed. For example, genomics and transcriptomics dataset may have tens of thousands of variables, and proteomics dataset may have only a few thousands, which may cause imbalance in the learning process. Overall, application of machine learning techniques to multiomics research will certainly be more frequent in coming years as a result of the expanding availability of biological data, including from the atherosclerotic plaque.

The development of machine learning methods to evaluate single-cell omics data has been a very active area of research in recent years due to the widespread deployment of single-cell omics technology and the large volume of produced data. In the review of current developments in machine learning techniques for studying single-cell transcriptomic and epigenomics data, Raimundo et al. [93] argue that a great majority of machine learning based tools have been simply imported from other disciplines with some aspects unsuited for biological data, hence reducing their performance. Additionally, it is still unclear whether machine learning models will be able to overcome the technical constraints that now exist for single cell genomics techniques, such as batch effects and dropouts. Hence, several conceptual and technical hurdles need to be resolved before machine learning becomes a useful tool aiding in the deconvolution of complex transcriptomics and multiomics datasets towards a deeper understanding of the molecular pathways gearing atherosclerosis development.

## 5 Future perspectives and conclusions

Characterization of atherosclerotic plaque represents an important step forward in the management of atherosclerotic cardiovascular disease. A better understanding of the complex interplay between various mechanisms within the plaque and the molecular changes that occur during disease progression may provide new targets for intervention and lead to the development of novel diagnostic and therapeutic tools, allowing for earlier detection and intervention.

Transcriptomic research has revealed novel players in the process of atherosclerosis. It has enhanced understanding of the cellular heterogeneity of the plaque, and has led to a better characterization of plaques. Further advancements in the protocols for bulk RNA sequencing deconvolution and scRNA-seq will deepen our knowledge of the plaque cellular complexity and develop more precise plaque categorisations that will pave the way to precision medicine approaches. A limitation of scRNA-seq is its inability to provide information about the spatial location of cells within a plaque. However, the development of spatial transcriptomics has enabled the high-throughput mapping of mRNA expression in tissues, including plaques, which enhance our understanding of the molecular mechanisms underlying plaque formation and progression. Moreover, the integration of transcriptomics data with other omics data can provide a more comprehensive picture of the molecular changes occurring in the plaque. To optimize outputs of multiomics strategies with machine learning approaches, reliable algorithms are needed, and this will require an understanding of how to incorporate the requirements of these algorithms into study design. For example, currently there are no recommendations on how to calculate study power when multiple omics analysis is planned. In addition, since risk factors such as age, sex, diabetes as well as environmental factors such as pollution and socio-demographic situation can have a significant impact on the overall disease phenotype, they should not be overlooked when designing studies on atherosclerosis.

Before being able to translate transcriptomics research findings to clinical application, there is an unmet need for thorough data validation and methodological harmonization. This applies to atherosclerosis as well as to any other biomedical research field. Thus, an important step is to create a research environment that facilitates collaboration, data sharing and harmonization. This will enable researchers to pool resources and expertise, and to conduct large-scale studies that are not feasible for individual researchers or institutions. By working together, scientists will be more equipped to overcome the challenges posed by the study of atherosclerotic plaque (complex multifactorial mechanism, low amount of material for transcriptomics analysis, low availability in human …). Moreover, the development of large-scale data sharing initiatives will allow researchers to access and analyse large amounts of data, leading to new discoveries and insights into plaque biology. Collaborative work is a leitmotiv of the members of the CardioRNA and AtheroNET COST Actions who drafted this manuscript.

## CRediT authorship contribution statement

All authors have equally contributed to the preparation of the manuscript. All authors have read and approved final version of the manuscript.

## Funding

Miron Sopić is funded the 10.13039/501100004564Ministry of Education, Science and Technological Development, Republic of Serbia through Grant Agreement with University of Belgrade-Faculty of Pharmacy No: 451-03-9/2021-14/200161, 10.13039/501100000780European Union (HORIZON-MSCA-2021-SE-01-01 - MSCA Staff Exchanges 2021 CardioSCOPE 101086397, HORIZON-MSCA-2021-PF- MAACS 101064175).

Dimitris Kardassis is supported by the General Secretariat for Research and Innovation of Greece (project code T2EDK-02361, MIS 5067590) which is implemented under the Action “RESEARCH - CREATE - INNOVATE” funded by the Operational Programme “Competitiveness, Entrepreneurship and Innovation” (NSRF 2014–2020) and co-financed by Greece and the 10.13039/501100000780European Union.

Lars Maegdefessel is supported by funding from the 10.13039/501100003793Swedish Heart-Lung- Foundation (20210450), the 10.13039/501100004359Swedish Research Council (Vetenkapsrådet, 201901577), a DZHK Translational Research Project on microRNA modulation in aortic aneurysms, the CRC1123 and TRR267 of the German Research Council (DFG), the 10.13039/100000002National Institutes of Health (NIH; 1R011HL150359-01), and the Bavarian State Ministry of Health and Care through the research project DigiMed Bayern.

Fabio Martelli is supported by the Italian Ministry of Health (“Ricerca Corrente”, RF-2019-12368521, The Italian Cardiology Network IRCCS RCR-2022-23682288, T4-AN-09 CAL.HUB.RIA), AFM-Telethon (# 23054), EU 10.13039/100010661Horizon 2020 (COVIRNA, Grant #101016072) and cardioRNA COST action (CA17129).

Ari Meerson is supported by a joint grant from MIGAL, Tel Hai Academic College and Ort Braude College, Israel.

Jelena Munjas is funded the 10.13039/501100004564Ministry of Education, Science and Technological Development, Republic of Serbia through Grant Agreement with University of Belgrade-Faculty of Pharmacy No: 451-03-9/2021-14/200161, 10.13039/501100000780European Union (HORIZON-MSCA-2021-SE-01-01 - MSCA Staff Exchanges 2021 CardioSCOPE 101086397).

Loredan Stefan Niculescu was funded by 10.13039/501100006730Romanian Ministry of Education and Research (#PN-III-P2-2.1-PED-2019-1897).

Monika Stoll acknowledges funding from the 10.13039/501100001659DFG (RTG2220 EvoPAD) and the European Union Marie Sklodowska-Curie grant agreement no. 813716 (TRAIN-HEART).

Paolo Magni is funded by 10.13039/100012352Universita’ degli Studi di Milano, Ministry of Health - Ricerca Corrente to IRCCS MultiMedica and project RF-2019-12370896, 10.13039/501100000780European Union (AtheroNET COST Action CA21153; HORIZON-MSCA-2021-SE-01-01 - MSCA Staff Exchanges 2021 CardioSCOPE 101086397).

Yvan Devaux is funded by the 10.13039/100010661EU Horizon 2020 project COVIRNA (Grant # 101016072), the 10.13039/501100001866National Research Fund (grants # C14/BM/8225223, C17/BM/11613033, COVID-19/2020-1/14719577/miRCOVID), CardioRNA COST action (CA17129), the 10.13039/501100004562Ministry of Higher Education and Research, and the Heart Foundation—Daniel Wagner of Luxembourg.

## Declaration of competing interest

The authors declare the following financial interests/personal relationships which may be considered as potential competing interests: Lars Maegdefessel is a scientific consultant and adviser for Novo Nordisk (Malov, Denmark), DrugFarm (Shanghai, China), and Angiolutions (Hannover, Germany), and received research funds from Novo Nordisk (Malov, Denmark) and Roche Diagnostics (Rotkreuz, Switzerland).
